# STAT3 mediates CAF-induced osimertinib resistance via regulating protein secretion in non-small cell lung cancer

**DOI:** 10.3389/fphar.2025.1546491

**Published:** 2025-07-09

**Authors:** Xuchen Fan, Sheng Wu, Honglong Wu, Yingying Huang, Xuhui Tong, Meiling Yu, Zhe Liu

**Affiliations:** ^1^ Department of Pharmacy, The First Affiliated Hospital of Bengbu Medical University, Bengbu, Anhui, China; ^2^ School of Pharmacy, Bengbu Medical University, Bengbu, Anhui, China; ^3^ Anhui Province Key Laboratory of Respiratory Tumor and Infectious Disease, The First Affiliated Hospital of Bengbu Medical University, Bengbu, Anhui, China

**Keywords:** tumor microenvironment, cancer associated fibroblasts, osimertinib resistance, signal transducer and activator of transcription 3, non-small cell lung cancer

## Abstract

**Introduction:**

EGFR-TKI resistance is an important factor limiting the clinical application of targeted drugs in NSCLC, but the mechanism remains unclear. The tumor microenvironment is the internal environment for cancer cells to survive, and it plays an important role in tumor resistance.

**Methods:**

In vitro assays: CCK-8 assay, wound healing, Transwell and Colony formation assay. Protein expression analyzed via Western blot. In vivo antitumor efficacy assessed by xenograft study. Target expression in tumors confirmed by immunohistochemical staining. Statistical analysis used t-test/ANOVA (p<0.05).

**Results:**

This study discovered that cancer associated fibroblasts (CAFs) in the tumor microenvironment induce Osimertinib resistance in NSCLC, and further study revealed that CAF-induced Osimertinib resistance in NSCLC is realized through its protein secretion. Interestingly, STAT3 is the key factor regulating CAF activation and secretion. Knockdown of STAT3 can block the secretory function of CAF, thereby reversing Osimertinib resistance in lung cancer. Furthermore, we blocked STAT3 activation in CAF with the novel STAT3 small molecule inhibitor LL1. LL1 effectively reversed CAF induced osimertinib resistance in NSCLC.

**Discussion:**

This project contributes to a deeper understanding of the molecular mechanism of tumor microenvironment mediated EGFR-TKI resistance in NSCLC, and provides theoretical basis and experimental data for the development of novel resistance reversal agents against the tumor microenvironment.

## Graphical Abstract

**Figure:**
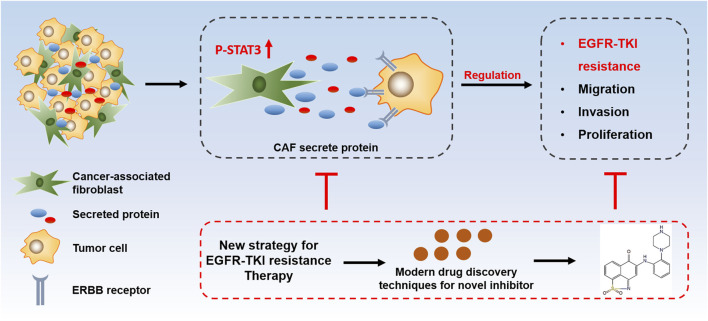
CAFs promoted osimertinib resistance via protein secretion in NSCLC.

## 1 Introduction

Lung cancer is a malignant tumor originated from the bronchial mucosa or glands of the lung. According to cancer statistics, the incidence rate and mortality rate of lung cancer ranked the first among malignant tumors in China (Sung et al., 2021). Non-small cell lung cancer (NSCLC) accounts for more than 80% of the incidence of lung cancer, and early surgery combined with radiotherapy results in high 5-year survival (Herbst et al., 2018; Hoy et al., 2019; Rodriguez-Canales et al., 2016). However, more than one-third of patients are in the middle or late stage when they are diagnosed in China, and about 75% of the patients with advanced disease have lost the chance of surgical treatment. Moreover, the effect of conventional radiotherapy is not satisfactory (Hong et al., 2015). EGFR-targeted drugs have significantly improved the therapeutic efficacy of NSCLC due to their high selectivity and low side effects (Garcia-Campelo et al., 2020; Kim et al., 2019; Remon et al., 2018). Although EGFR-TKIs have brought significant clinical benefits to NSCLC patients, physicians have to face another thorny problem: most patients develop drug resistance after EGFR-TKIs treatment (Mu et al., 2020; Shu et al., 2020; Sun et al., 2020). Therefore, clarifying the mechanism of EGFR-TKI resistance is urgent in the field of tumor pharmacology.

Although genetic mutation especially in first- and second-generation EGFR-TKIs is one of the most important causes of EGFR-TKI resistance, the variety and uncontrollability of mutations have led to a stalemate in drug resistance research. In fact, the mutation rate of EGFR-C797S, an important gene for third-generation EGFR-TKI resistance, is only 15%, which means that there is no relatively stable and high percentage of resistance mutations for osimertinib (Thress et al., 2015; Wu Z. et al., 2021). The resistance mechanisms of third-generation EGFR-TKIs are primarily categorized into EGFR-dependent and EGFR-independent mechanisms. EGFR-dependent mechanisms are mainly characterized by multiple EGFR gene mutations, including T790M and C797S mutation. EGFR-independent mechanisms refer to the activation of bypass signaling pathways (MET/HER2 amplification), histological transformation (conversion to small cell lung cancer), and activation of alternative compensatory signaling pathways. To address this challenge, combination therapeutic strategies are currently under investigation, such as chemotherapy or radiotherapy. However, existing findings exhibit significant limitations and uncertainties, necessitating further in-depth exploration (Li S. et al., 2022). Tumor microenvironment refers to the surrounding microenvironment in which tumor cells exist, including blood vessels, immune cells, fibroblasts, bone marrow-derived inflammatory cells, various signaling molecules, and extracellular matrix and other components of tumor tissues (Arneth, 2019). Tumor microenvironment is a complex environment for tumor cells to survive and develop, and the various cells within the microenvironment play important roles in tumor development and drug resistance (Altorki et al., 2019). In fact, it has been reported in the literature that the microenvironment influences the mechanisms of tumor drug resistance: (1) the tumor microenvironment secretes pro-oncogenic substances to induce drug resistance; CAF-derived conditioned media was observed to induce breast cancer cell growth and radioresistance. CAFs secrete interleukin 6 (IL-6) which activates signal transducer and activator of transcription 3 (STAT3) signaling pathway, thus promoting the growth and radioresistance of breast cancer cells. Treatment with an inhibitor of STAT3 or an IL-6 neutralizing antibody blocked the growth and radioresistance induced by CAFs (Guo et al., 2023). (2) the diversity of tumor stromal cell components leads to insensitivity to drugs; and (3) the tumor microenvironment forms a physiological barrier to prevent drugs from penetrating into the tumor tissues and promotes proliferation of tumors (Hinshaw and Shevde, 2019; Wang et al., 2018; Wu and Dai, 2017).

Considering the resistance mechanism of EGFR-TKIs require continuous stimulatory signals or environment. The characteristics of tumor microenvironment precisely meet this requirement. It provides a continuous “stable environment” which results in the continuous activation of pro-oncogenic signals for EGFR-TKI resistance. Currently, the researches about tumor microenvironment mainly focuses on immunity and metastasis, but the mechanism related to drug resistance is still unclear. Although multiple STAT3 inhibitors have entered clinical trials, their efficacy remains limited with significant side effects. For instance, OPB-31121 was terminated in Phase II trials due to gastrointestinal toxicity and hepatotoxicity (Yang et al., 2023). Additionally, STAT3’s physiological roles in normal tissues may lead to risks of immune suppression or dysregulation of inflammatory responses caused by the inhibitors (Ji et al., 2025). Herein, the objective of this study was to determine whether tumor microenvironment promoted EGFR-TKI resistance in lung cancer, and to delineate the possible mechanism.

## 2 Materials and methods

### 2.1 Reagents and chemicals

Osimertinib (HY-15772) were purchased from MedChemExpress, USA. RPMI 1640 and DMEM were purchased from Gibco, United States. Fetal Bovine Serum (164210-50) was purchased from Pricella, China. Cell Counting Kit-8 (CCk-8) was purchased from Biosharp, China. Penicillin-Streptomycin Solution, 100X was purchased from Beyotime, China.

### 2.2 Cell culture

Human lung cancer PC-9 and HCC827 cell lines and human lung CAFs were purchased from Wuhan Pricella Biotechnology. The PC-9 and HCC827 cell lines were cultured in RPMI 1640 supplemented with 10% fetal bovine serum, 100U/mL penicillin and 0.1 mg/mL streptomycin. CAFs was cultured in DMEM supplemented with 10% fetal bovine serum, 100U/mL penicillin and 0.1 mg/mL streptomycin. These cells are maintained in a standard incubator at 37°C with 5% CO_2_ to support cell growth and proliferation, and passaged when reaching approximately 80% confluence, and the medium was changed every other day.

### 2.3 Construction of osimertinib-resistant cell lines

The NSCLC cell line PC-9 and HCC827 was induced by a combination of intermittent high-dose shock and gradient incremental approach, and the cells collected during the logarithmic growth phase were spread on 6-well cell culture plates at 5 × 10^5^ cells per well and cultured for 24 h. Firstly, the half inhibition concentration (IC50) of the cell line was measured by CCK8 method, and the drug-free medium was changed to the medium containing 100 nmol/L osimertinib for 12–24 h. The cell status was observed. If there were a large number of floating dead cells at the initial stage, the medium was promptly replaced with a concentration of IC50 (45 nmoI/L) to continue the culture until the surviving cells resumed normal proliferation. When the cells grew well in the medium containing 45 nmol/L osimertinib and were successively passaged for more than 3 times, the cells were then shock-cultured with medium containing 100 nmol/L osimertinib for 12–24h, after which they were replaced with medium containing 45 nmol/L osimertinib (the above is a culture cycle). When the cells experienced three cycles and were in a more stable state, the drug concentration could be elevated according to the drug concentration difference of 50 nmol/L, acted for 12–24 h, observed the cell state, and replaced with the medium containing 45 nmol/L osimertinib for another 24–72 h. When the density of adherent cells in the drug-containing medium was lower than 20%–30% or a large number of floating dead cells appeared. The drug withdrawal treatment was taken in time, and the cells could be stabilized and proliferated in the drug-containing environment after several times of drug administration. When the drug screening concentration reached 5 times the IC50, it was elevated by a concentration difference of 100 nmol/L, and the amount of the drug in the medium was adjusted according to the cell status until the cells were able to grow normally at the set concentration. The test took a total of 7 months, and the established drug-resistant cell line was named PC-9-R/HCC827-R.

### 2.4 CCK-8 assay

The PC-9 and HCC827 cells were prepared as single-cell suspensions, and seeded into 96-well plates at the density of 5 × 10^4^ cells/mL. The cells were cultured in a 5% CO_2_ incubator for 24 h. After 24 h, 10 μL of CCK-8 was added to each well, and the cells were incubated for 1 h in the dark. The absorbance (A) value was detected at 450 nm wavelength using a plate reader. All the experiments repeated three times.

### 2.5 Wound healing

The PC-9, HCC827 cells were seeded at the density of 4 × 10^5^ cells/mL into 6-well plates, and cultured for 24 h. A vertical scratch was made with a 100 μL pipette tip. The supernatant was discarded, and the cells were washed three times with PBS. Observations and photographs were taken under an inverted microscope at 0, 24, or 48 h after treatment. The scratch distance was analyzed using ImageJ software, and the cell migration rate was calculated using Graphpad Prism 9.0 software. The cell migration rate = (scratch distance at 0 h-scratch distance at 24 or 48 h)/scratch distance at 0 h × 100 %. The experiment was repeated three times.

### 2.6 Transwell

The chambers were placed into 24-well plates, and coated with 50 μL of matrigel basement membrane matrix at the bottom of the polycarbonate membrane. Incubate for 2 h at 37°C to form the gel. The cells were seeded at the density of 1 × 10^6^ cells/mL into the upper chamber of the well (200 μL per well), and 600 μL of complete medium was added to the lower chamber. After incubating for 24 h, the cells were treated with osimertinib, LL1 or combination for another 24 s. Then the chambers were fixed with 4% polyformaldehyde for 30 min, and stained with crystal violet for 15 min. The number of cells that passed through the chamber was observed and photographed under a microscope. The experiment was repeated three times.

### 2.7 Colony formation assay

HCC827 and PC-9 cells were seeded at the density of 3 × 10^3^ cells/mL on six well plates. After 24 h incubation, the cells were washed twice with PBS, and treated with osimertinib, LL1 or combination. The medium was renewed every 2 days, and the cells were continuously cultured for 7–12 days. Then the cells were fixed with methanol for 30 min, and stained with crystal violet for 3 min. The experiment was repeated three times.

### 2.8 Western blot

The cells were centrifuged and precipitated, and lysed by lysis solution and 1% protease inhibitor. Protein concentration was determined using Micro BCA Protein Assay kit (Beyotime Biotechnology, China). Samples were electrophoresed on decolorized SDS-page gels after being heated to 99°C for 5 min in the upwelling buffer. Proteins were transferred to polyvinylidene fluoride (PVDF) membranes and closed with rapid closure solution for 30 min. The membranes were blocked by 5% skim milk in TBS with 0.1% of Tween-20 (TBST) for 2 h at room temperature prior to be incubated overnight with primary antibodies. The following antibodies were used in this study: STAT3 (ab68153, Abcam, USA), α-SMA (#19245, Cell Signailing, USA), FAP (#52818, Cell Signailing, USA), Vimentin (#5741, Cell Signailing, USA) and p-STAT3 (#9145, Cell Signaling, USA). The membranes were than washed thrice in TBST and incubated with horseradish peroxidase-conjugated secondary antibodies for 1 h. The β-actin and secondary antibodies were purchased from Beyotime. After consecutive washes, the membranes were visualized using a chemiluminescence kit (PerkinElmer, USA).

### 2.9 Transfection

The siRNAs targeting STAT3 were obtained from GenePharma (Shanghai, China), with the following sequences: NC, TTC​TCC​GAA​CGT​GTC​ACG​T; STAT3-Homo-989, TTC​AGA​CCC​GTC​AAC​AAA​TTA; STAT3-Homo-1297, CTC​AGA​GGA​TCC​CGG​AAA​TTT; STAT3-Homo-1762, TTG​GGA​CCT​GGT​GTG​AAT​TAT. A negative control targeting no specific sequences was used for comparison. CAFs were seeded at a density of 2 × 10^5^ cells per well in 6-well plates and cultured for an additional 24 h. Infect the cells using lentiviruses, The fluorescence was observed following 48 h Infection. The knockdown efficiency of STAT3 was confirmed through Western blotting.

### 2.10 Immunohistochemical

The sections were sequentially placed in xylene I for 15 min-xylene II for 15 min-xylene III for 15 min-anhydrous ethanol I for 5 min-anhydrous ethanol II for 5 min-85% alcohol for 5 min-75% alcohol for 5 min-washed in distilled water. Antigen repair was performed in citric acid in high heat for 10 min cease fire for 8 min to medium-low heat for 8 min. A certain proportion of KI67, p-STAT3 and STAT3 primary antibody was added dropwise to the sections and incubated flat in a wet box at 4°C overnight. The slides were washed and the secondary antibody of the corresponding species was added dropwise to the primary antibody. Then the slides were washed and freshly prepared DAB color solution was added dropwise, and the positive color was brownish yellow. Nuclei were restained with hematoxylin and finally dehydrated and sealed.

### 2.11 *In vivo* xenograft study

Seven-week BALB male mice were randomized to eight mice in each group, which were divided into PC-9 group, PC-9 + CAF group, PC-9 treated with osimertinib group (5 mg/kg, once every 2 days), PC-9 + CAF treated with osimertinib group (5 mg/kg, once every 2 days) and PC9 + CAF treated with osimertinib + LL1 (5 mg/kg, once every 2 days) group. The body weights of mice were recorded daily and the average tumor volume in each group was expressed in mm^3^ and calculated according to the equa-tion (lenth × width^2^)/2. At the end of the study, tumor xenografts were removed and weighed. Obtained tumor samples were quickly frozen in liquid nitrogen and stored at −80°C for immunoblot analysis.

### 2.12 Statistical analysis

All data are presented as mean ± SD. An unpaired two-tailed Student’s t-test was used to compare the difference between two groups, while for multiple comparisons, one-way ANOVA followed by Tukey’s correction was employed. *P* values for tumor growth curves were calculated using a two-way ANOVA. A *P* value <0.05 was considered statistically significant, denoted as *P < 0.05, **P < 0.01. All statistical analyses were conducted using Prism version 9.0 (GraphPad). Unless indicated otherwise, experiments were carried out at n = 3, where n = number of independent experiments.

### 2.13 Supporting materials and methods

For details regarding proteomics analysis, bioinformatics analysis, and other related procedures refer to the Supplementary Material.

## 3 Results

### 3.1 CAF induces osimertinib resistance of NSCLC

**FIGURE 1:**
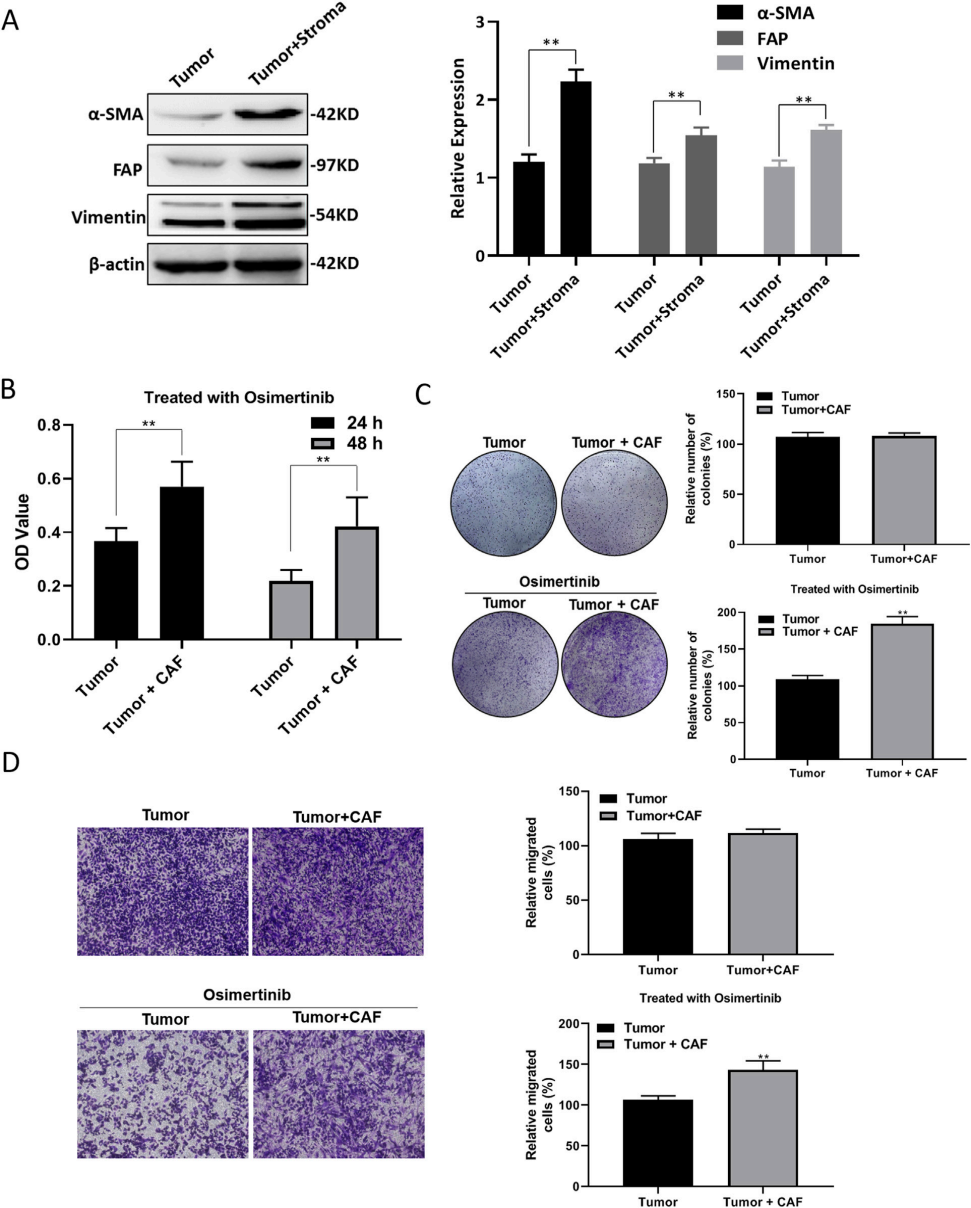
CAF induces osimertinib resistance of NSCLC. **(A)** Western blotting showed the level of α-SMA, Vimentin and FAP in patient-derived tumor with stroma tissue and tumor tissue. **(B)** CCK-8 assay showed the effect of CAF on the cell proliferation of tumor following the treatment of osimertinib. **(C)** Colony formation assay showed the effect of CAF on the single cell proliferation potential of tumor cells following the treatment of osimertinib. **(D)** Transwell assay showed the effect of CAF on the invasive ability of tumor cells. Data are presented as mean ± standard deviation. *P* values were calculated using unpaired two-tailed Student’s t-test. **P < 0.01.

In order to investigate the relationship between tumor microenvironment and EGFR-TKI resistance in lung cancer, we tested the expression levels of tumor tissue markers in lung cancer patients with drug resistance. We found that the expression of α-SMA, FAP and Vimentin were significantly elevated in the tumor tissues mixed with stromal cells (Figure 1A). The results indicated that the stromal cells may mainly consist of cancer-associated fibroblasts (CAFs). We purified and separated CAF and tumor cell, and found that co-culture of CAF and tumor cell could reduce the sensitivity of lung cancer cells to osimertinib (Figures 1B,C). Moreover, the results of transwell assay demonstrated that the CAFs could promote the migration of lung cancer cells under the stimulation of osimertinib (Figure 1D). The above results suggest that the CAFs in the tumor microenvironment can promote the resistance of lung cancer cells to osimertinib.

### 3.2 CAF induces osimertinib resistance in PC-9 and HCC827 lung cancer cells

**FIGURE 2:**
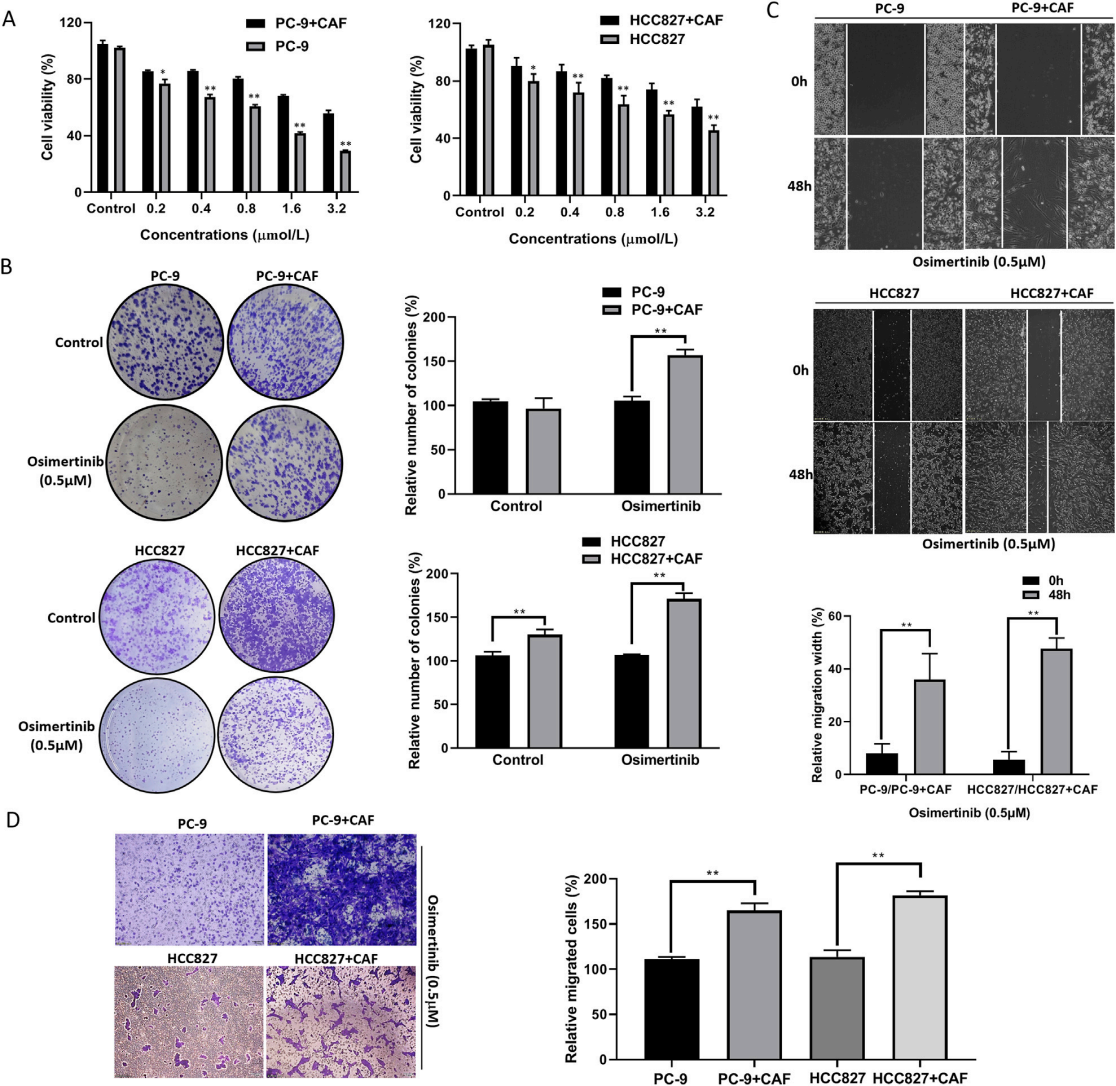
CAF induces osimertinib resistance in PC-9 and HCC827 lung cancer cells. **(A)** CCK-8 assay showed the effect of CAF on the cell proliferation of PC-9 and HCC827 cells. **(B)** Colony formation assay showed the effect of CAF on the single cell proliferation potential of PC-9, and HCC827 cells. **(C)** Wound healing assay showed the effect of CAF on the migration of PC-9, and HCC827 cells. **(D)** Transwell showed the effect of CAF on the invasion of PC-9, and HCC827 cells. Data are presented as mean ± standard deviation. *P* values were calculated using unpaired two-tailed Student’s t-test. **P < 0.01.

To investigate the effect of CAF on osimertinib resistance in lung cancer cells, we induced resistance to osimertinib in PC-9 and HCC827 cells. Co-culture of CAF with PC-9 or HCC827 cells significantly shortened the time for induction of resistance in lung cancer cells (Supplementary Figure S1A). Compared with primary cells, the proliferation and colony formation ability of resistant cells were significantly increased (Supplementary Figures S1B, 1C). Moreover, resistant cells showed enhanced invasive and migration ability (Supplementary Figures S1D, 1E). Further studies revealed that the CAFs significantly increase the proliferation and colony formation ability of PC-9 or HCC827 cells (Figures 2A,B). The CAFs enhanced invasive and migration ability in PC-9 or HCC827 cells (Figures 2C,D). These results demonstrated that co-culture of CAF with lung cancer cells exhibited similar properties to osimertinib resistance lung cancer cells. More importantly, the CAF stimulated increased osimertinib resistance in lung cancer cells (Figures 2A–D).

### 3.3 CAF may induce osimertinib-resistance in lung cancer cells by secreting proteins

**FIGURE 3:**
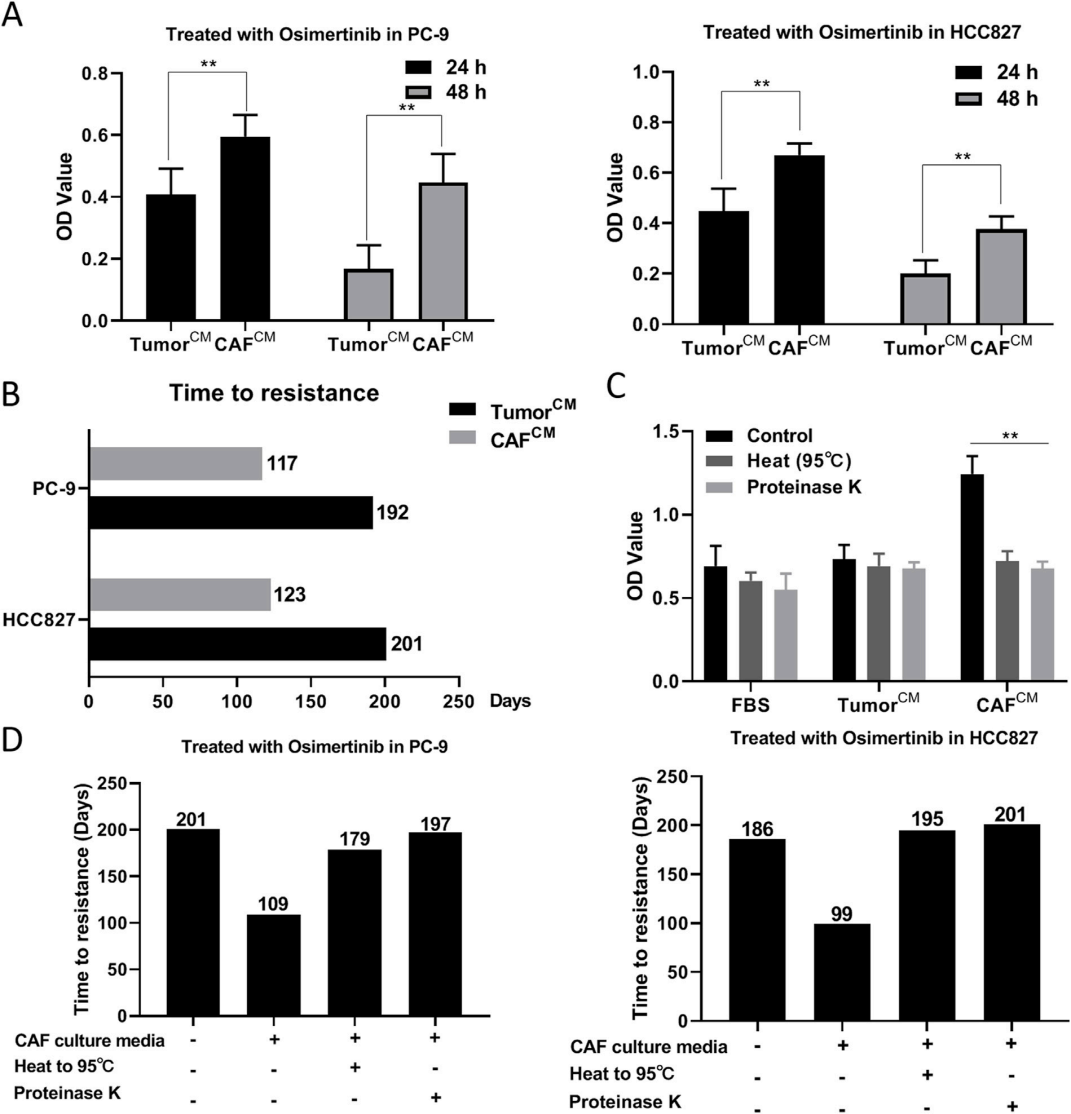
CAF may induce osimertinib resistance in lung cancer cells by secreting proteins. **(A)** CCK-8 assay showed the effect of CAF-derived conditioned medium on the cell proliferation of PC-9 and HCC827 cells. **(B)** Effect of CAF-derived conditioned medium on the time to induce osimertinib resistance in PC9 and HCC827 cells. **(C)** Protease K or heating to 95° inhibited CAF-derived conditioned medium induced osimertinib resistance in PC-9 and HCC827 cells. **(D)** Protease K or heating to 95° increased the time for CAF-derived conditioned medium to induce osimertinib resistance in PC-9 and HCC827 cells. Data are presented as mean ± standard deviation. *P* values were calculated using unpaired two-tailed Student’s t-test. **P < 0.01.

CAF has the characteristics of rapid proliferation, aggressive migration and high secretion. Considering that CAF are more metabolically active and produce more secretory substances than normal fibroblasts, we sought to explore the relationship between CAF secretions and osimertinib resistance in lung cancer cells. We added CAF conditioned medium into PC-9 and HCC827 cell culture environment, and the results showed that CAF conditioned medium could reduce the osimertinib sensitivity of PC-9 and HCC827 cells (Figure 3A). Moreover, CAF conditioned medium significantly reduced the time to induce osimertinib resistance in PC-9 and HCC-827 lung cancer cells (Figure 3B). In order to determine the nature of CAF secretions, we heated CAF conditioned medium to 95°C or added proteinase K treatment. We found its induction of drug resistance in lung cancer cells was significantly reduced (Figure 3C). In addition, heating or proteinase K significantly increased the time for CAF supernatant to induce osimertinib resistance in lung cancer cells (Figure 3D). The above results suggest that CAF may induce osimertinib resistance in lung cancer cells by secreting proteins.

### 3.4 Proteomic analysis of CAF secreted proteins

**FIGURE 4:**
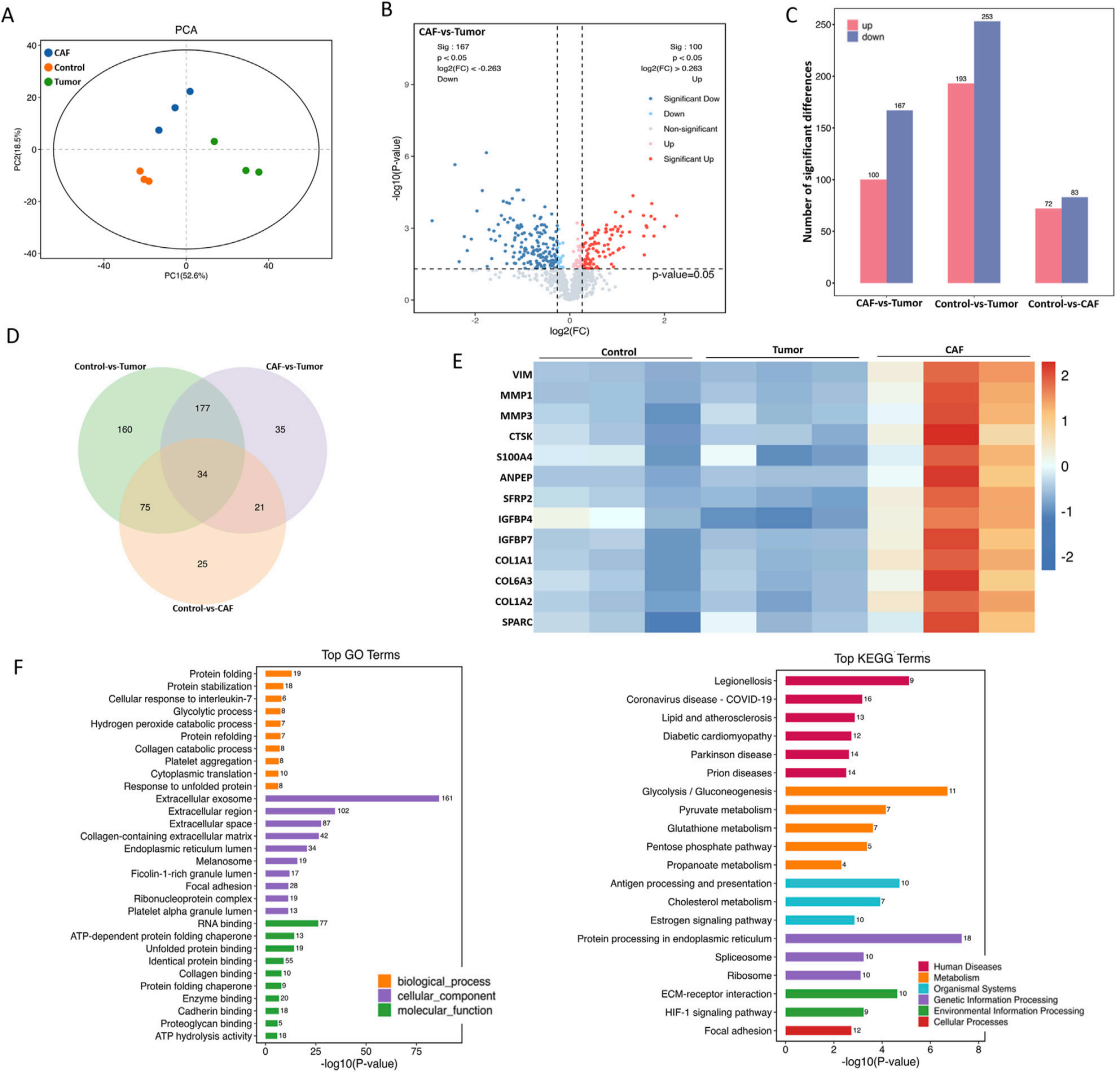
Proteomic analysis of CAF secreted proteins. **(A)** Total protein extracted from CAF-derived conditioned medium or Tumor-derived conditioned medium were subjected to proteomic synthesis and analyzed by the mass spectrum. PCA plot showing gene clusters from CAF and Tumor samples. **(B)** Volcano plot shows all genes with measured expression, dotted black vertical lines indicate log2 FC of one and -1, and the dotted black horizontal line indicates a p-value of 0.05. Genes significantly upregulated in CAF compared to Tumor are shown in red, significantly downregulated genes are shown in blue. **(C)** Differential expression secreted protein of CAF and tumor. **(D)** The intersection targets of tumor against CAF (Venn diagram). **(E)** The heat map shows the major upregulated genes (marked in red) and the downregulated genes (marked in blue) associated with the development of tumor resistance. **(F)** GO and KEGG enrichment analysis of the differential expression secreted proteins of CAF and tumor.

To further explore the mechanism of CAF-induced resistance, we analyzed the protein composition of CAF and tumor cell conditioned medium by proteomics. There were significant differences among the three groups, with PC1 and PC2 contributing 52.6% and 18.5%, respectively (Figure 4A). Furthermore, the CAF group and the tumor group were significantly separated (Figure 4B). A total of 267 proteins were differentially expressed in the CAF group compared with the tumor group, of which 100 proteins were upregulated and 167 proteins were downregulated (Figure 4C). Compared with tumor group and medium group, there were 55 common differentially expressed proteins analyzed by venn (Figure 4D). In addition, the results of protein expression heat map showed that Vim, MMP-3 and MMP-1 were the most significantly different among CAF secreted proteins (Figure 4E). The results of GO enrichment analysis showed that the functions of CAF differentially expressed proteins were mainly enriched in RNA binding, glycolysis, exocytosis, and protein binding (Figure 4F). The above results indicate that CAF secrete a variety of pro-oncogenic proteins, and these proteins may be related to the osimertinib resistance induced by CAF.

### 3.5 STAT3 regulates CAF-induced osimertinib resistance in PC-9 and HCC827 cells

**FIGURE 5:**
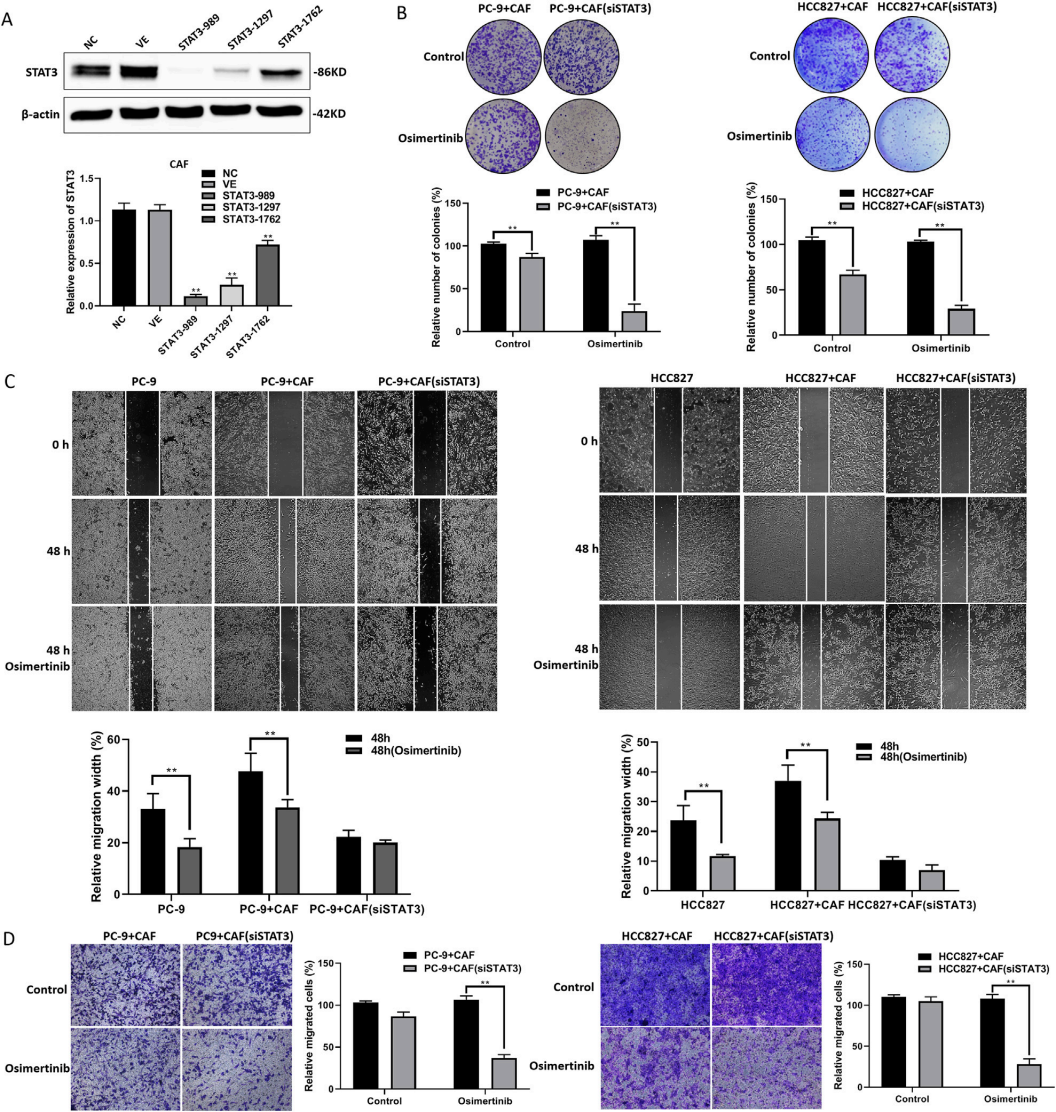
STAT3 regulates CAF-induced osimertinib resistance in PC-9 and HCC827 cells. **(A)** The STAT3 stable knockdown of CAF were verified using Western blotting. **(B)** Colony formation assay showed downregulation of STAT3 blocks the effect of CAF on the proliferation of PC-9 and HCC827 cells following the treatment of osimertinib. **(C)** Wound healing assay showed downregulation of STAT3 blocks the effect of CAF on the migration of PC-9 and HCC827 cells following the treatment of osimertinib. **(D)** Transwell showed downregulation of STAT3 blocks the effect of CAF on the invasion of PC-9 and HCC827 cells following the treatment of osimertinib. Data are presented as mean ± standard deviation. *P* values were calculated using unpaired two-tailed Student’s t-test. **P < 0.01.

We found that the expression of p-STAT3 was significantly elevated in CAF compared with “PAF” (para-cancer fibroblasts) (data not shown). STAT3 activation may be a critical target in CAF-induced osimertinib resistance in lung cancer cells. We constructed a CAF cell line with stable knockdown of STAT3 via lentivirus (Figure 5A). We found that knockdown of STAT3 expression significantly attenuated CAF-induced osimertinib resistance in colony formation assay (Figure 5B). Moreover, we found the same phenomenon in lung cancer cell migration and invasion (Figures 5C,D). The above results suggest that STAT3 may be critical to CAF-induced osimertinib resistance in lung cancer cells. Furthermore, the results of protein array showed that the expression levels of MMPs (Especially MMP-3) were significantly decreased after knockdown of STAT3 (Supplementary Figure S2). Notably, the proteomic results suggested increased secretion of MMP-1, MMP-2 and MMP-3 in CAF. These results combined with protein array demonstrated that CAF may regulates lung cancer resistance through the STAT3/MMP signaling pathway, and inhibition of STAT3 in CAF can block the secretion of MMP which induce osimertinib resistance in lung cancer cells. In addition, KEGG enrichment analysis showed that the downregulated genes after STAT3 silencing were mainly involved in signaling pathways related to EGFR tyrosine kinase inhibitor resistance (Supplementary Figure S3).

### 3.6 STAT3 inhibition blocks CAF-induced osimertinib resistance *in vivo*

**FIGURE 6:**
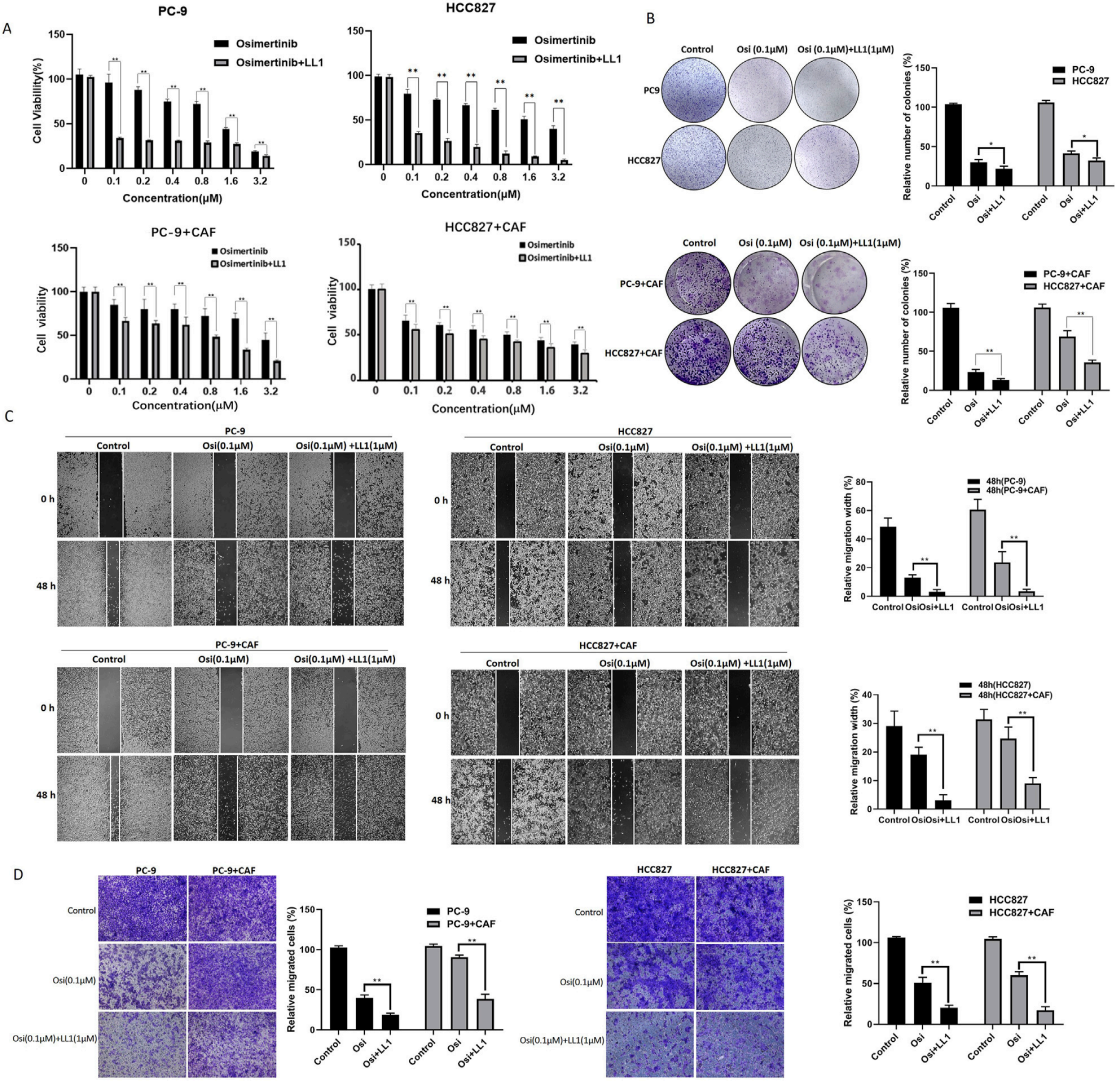
LL1 inhibits CAF-induced osimertinib resistance in PC-9 and HCC827 cells. **(A)** CCK-8 assay showed LL1 inhibits the effect of CAF on the proliferation of PC-9 and HCC827 cells following the treatment of osimertinib. **(B)** Colony formation assay showed LL1 inhibits the effect of CAF on the proliferation of PC-9 and HCC827 cells following the treatment of osimertinib. **(C)** Wound healing assay showed LL1 inhibits the effect of CAF on the migration of PC-9 and HCC827 cells following the treatment of osimertinib. **(D)** Transwell showed LL1 inhibits the effect of CAF on the invasion of PC-9 and HCC827 cells following the treatment of osimertinib. Data are presented as mean ± standard deviation. *P* values were calculated using unpaired two-tailed Student’s t-test. **P < 0.01.

**FIGURE 7:**
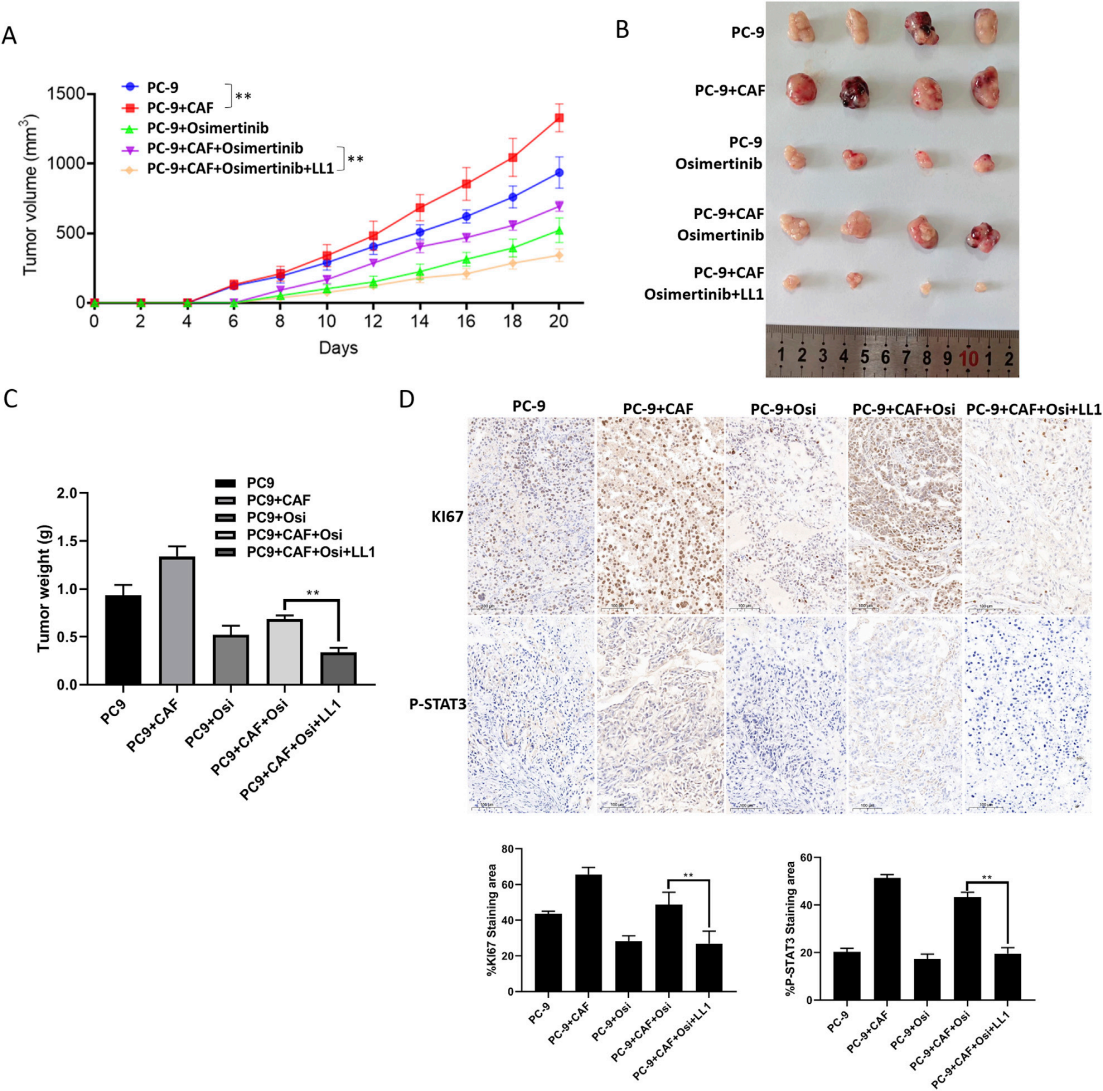
STAT3 inhibition blocks CAF-induced osimertinib resistance *in vivo*. **(A)** The growth curve of PC-9 tumors (n = 8) and PC-9 plus CAF (n = 8). **(B)** General photograph of xenograft at the 20th day after injection of PC-9 or PC-9 plus CAF into subcutanea posterioris axilla with or without osimertinib and LL1. **(C)** The weight of xenograft at the 20th day. **(D)** IHC analysis showing the protein expression of Ki67 and p-STAT3 in tumor tissues. Scale bar, 100 μm. Scale bars: 100 μm. Data are presented as mean ± standard deviation. *P* values were calculated using unpaired two-tailed Student’s t-test or two-way ANOVA for Sidak’s multiple-comparisons test. *P < 0.05, **P < 0.01.

We have found that STAT3 activation is critical for CAF-induced osimertinib resistance in lung cancer through the above data. Thus, inhibiting STAT3 activation may be a new strategy to block CAF protein secretion to reverse osimertinib resistance in lung cancer. LL1 is a novel STAT3 inhibitor, which can selectively inhibit p-STAT3 expression. We treated PC-9 and HCC827 cells with LL1, and the results of CCK-8 assay demonstrated that LL1 reversed CAF-induced osimertinib resistance in PC-9 and HCC827 lung cancer cells (Figure 6A). Moreover, the reverse effect of LL1 could also be observed in colony formation, migration and invasion (Figures 6B–D). In order to investigate the effect of LL1 on lung cancer cell resistance *in vivo*, we constructed xenograft model to test it. We found that CAF promotes tumor growth, and CAF induces tumor resistance to osimertinib *in vivo* (Figure 7A). The results showed a significant reduction in the volume of tumors following the treatment of LL1 plus osimertinib compared to osimertinib alone (Figures 7A,B), suggesting that LL1 could reverse CAF-induced osimertinib resistance of lung cancer cells *in vivo*. In addition, CAF markedly enhanced PC-9 xenograft growth, whereas osimertinib treatment significantly inhibited tumor development. In CAF-conditioned tumors, osimertinib’s antitumor activity was partially blunted, but the addition of LL1 fully restored sensitivity and achieved the most pronounced tumor suppression (Figure 7C). No significant changes in body weights or obvious signs of toxicity, such as loss of appetite, decreased activity, or lethargy, were observed (Supplementary Figure S4). Immunohistochemical results showed that CAF could promote elevated levels of KI67 and p-STAT3 in tumor, which was reversed by LL1 in combination with osimertinib (Figure 7D). Furthermore, haematoxylin and eosin staining of the heart, liver, spleen, lung, and kidney demonstrated that LL1 plus osimertinib had no damage of the major organs (Supplementary Figure S5). In conclusion, LL1 could reverse CAF-induced osimertinib resistance of NSCLC *in vivo*.

## 4 Discussion

Lung cancer treatment has entered the era of precision therapy, and targeted drugs with good efficacy and low side effects have gradually replaced traditional chemotherapeutic drugs as the first-line drugs. Doctors can determine the types of tumor-related gene mutations and select suitable targeted drugs through genetic testing. EGFR mutation is one of the most prevalent types of gene mutation in lung adenocarcinoma patients. The EGFR-TKI-sensitive mutation rate of Europeans was about 10%, however the non-smoking Asians was as high as 40% (Rivera and Wakelee, 2016). Although the first- and second-generation EGFR-TKIs are superior to traditional chemotherapeutic drugs in terms of objective remission rate and side effects, the emergence of drug resistance is a critical reason that restricts the clinical application of EGFR-TKIs.

Previous studies have shown two main mechanisms of resistance to first- and second-generation EGFR-TKIs: (1) T790M mutation in the amino acid fragments bound by EGFR-TKIs, which accounts for 50% of the cases; (2) bypass activation to compensate for the inhibition of the EGFR pathway, such as the signaling pathways of c-Met, HER2, and KRAS, etc (Liu B. et al., 2020; Nishiyama et al., 2020; Onitsuka et al., 2010; Park et al., 2019; Yoon et al., 2019). In addition, third-generation EGFR-TKIs, osimertinib had solved the problem of T790M mutation resistance, and possessed of better selectivity at the same time. According to the National Comprehensive Cancer Network (NCCN) of 2021, osimertinib was listed as first-line treatment of NSCLC. However, more and more reports about osimertinib resistance have gradually increased followed by the frequent use of osimertinib in the clinic. According to the current research, the mechanism of osimertinib resistance was divided into four categories: downstream gene mutation or amplification, histological transformation, bypass activation, and unexplained resistance mechanisms (Jiang et al., 2021; Zeng et al., 2022; Zhu et al., 2021). It can be seen that the phenomenon of bypass pathway over-activation exists in all generation osimertinib resistance. Our data showed that tumor tissues mixed with stromal cells were significantly less sensitive to osimertinib compared with tumor tissues in which stromal cells were excluded, suggesting that the presence of stromal cells may protect tumors from being killed by osimertinib. This finding reminds us that certain components of tumor microenvironment may play an important role in osimertinib resistance.

We found that the addition of CAF conditioned medium to the culture environment of lung cancer cells protected the cells from being killed by osimertinib. Furthermore, our results demonstrated that some certain proteins in the supernatant of CAF induces osimertinib resistance in lung cancer. The molecular biological functions and biological processes of CAF secretory proteins were enriched for binding function, extracellular matrix organization, and further proteomic analysis revealed that Matrix Metalloproteinase (MMPs) and Neuregulin-1 (NRG1) in CAF secretion signal was abnormally elevated. The extracellular matrix (ECM) is a complex network of biomolecules in the extracellular region that provides structural and mechanical support to surrounding cells, and it makes a significant contribution to the development of cancer (Huang et al., 2021). CAF is the major producer of ECM. During the transition from a normal ECM phenotype to a cancerous ECM phenotype, the ECM undergoes remodeling of growth factors and enzymes levels, and tumor ECM develops more fibrosis and stiffness (Fan et al., 2021). In addtion, activated CAF increases MMPs secretion to regulate the stiffness of ECM, thereby inducing tumor resistance (Qin et al., 2020). Study shows that upregulation of MMP-2 in the tumor microenvironment can lead to vemurafenib resistance in melanoma (Sandri et al., 2016). Previous research reported that ERBB3 was significantly activated in EGFR-TKI-resistant, while NRG1 was a direct ligand of ERBB3 receptor which promotes cell growth and differentiation via interacting with ERBB3. The above results suggested that CAF in the tumor microenvironment may activate the ERBB3 signaling pathway through the secretion of NRG1 to induce osimertinib resistance in NSCLC. NRGs which belong to the epidermal growth factor family were a group of signaling proteins that mediate cell-cell interactions. NRG1 mainly binds to ERBB3 heterodimer, then activates various intracellular signaling cascades, such as PI3K/Akt, JAK-STAT3, RAS, Erk1/2, etc. In addition, NRG1 has been shown to be a marker of cetuximab resistance in colorectal cancer (Luraghi et al., 2018). Thus, inhibiting the function of the secreted protein NRG1 may be the key to reverse osimertinib resistance. However, the cellular biology of NRG1 is complex, and direct targeting of NRG1 may cause serious adverse effects. Therefore, we attempted to find a way to reverse drug resistance by targeting the tumor microenvironment.

Normal fibroblasts are inhibitory to tumorigenesis development. Therefore, cancer need to reprogram normal fibroblasts into CAFs (Chhabra and Weeraratna, 2023; Sizemore et al., 2018). The fibroblasts are normally quiescent, and they will be activated and transformed into myofibroblasts during wound healing or tissue fibrosis. This process will lead to tissue repair and scar formation (Dzobo and Dandara, 2020; Sahai et al., 2020). Studies have shown that activated CAFs exhibit enhanced proliferative and migratory properties compared to quiescent fibroblasts. In addition, CAFs can secrete large amounts of growth factors, pro-inflammatory cytokines, and chemokines to promote tumor growth and drug resistance (Baschieri et al., 2023). Tumors have been regarded as ‘unhealable wounds’ for a long time, and this notion suggests that targeting CAF activation has great potential for cancer treatment. Studies have shown that exposure of fibroblasts to tumor inflammation triggers epigenetic transitions mediated by histone acetyltransferases and DNA methyltransferases, leading to sustained activation of STAT3 which is critical for the maintenance of CAF phenotype and function (Liu Q. et al., 2020; Ma et al., 2019). Furthermore, cytokines or inflammatory factors secreted by cancer cells and stromal cells shall induce CAF activation by stimulating STAT3 activation. More and more evidences demonstrated that the expression of p-STAT3 in CAF is negatively correlated with the prognosis in colorectal cancer, and the persistent activation of STAT3 in CAF promotes the tumor growth and drug resistance in colorectal cancer (Heichler et al., 2020; Kuzet and Gaggioli, 2016). Further knockdown experiments revealed that downregulation of STAT3 could inhibit the expression of NRG1, MMP-1, MMP-2 and MMP-3. Moreover, downregulation of STAT3 in CAF weakened the effect of CAF-induced osimertinib resistance. The results indicated that the expression of STAT3 regulates the secretion of CAF, and the inhibition of STAT3 may reverse CAF-induced osimertinib resistant in NSCLC.

Despite the fact that STAT3 has been regarded as a classical oncogene and inhibition of STAT3 activation can significantly reverse drug resistance, there are still no STAT3 small molecule inhibitors that are FDA-approved. In recent years, several studies of small molecule inhibitors of STAT3 have been reported, such as BP-1-102, BBI-608, Stattic and HO-3867, etc (Froeling et al., 2019; Rath et al., 2014; Yue et al., 2024). However, there are still several challenges in the research and development of STAT3 inhibitors: high *in vivo* toxicity, weak selectivity and low durability. Therefore, high efficiency and low toxicity STAT3 inhibitors are still the direction of future research. Studies have shown that STAT3 is transiently activated in normal tissues to maintain normal cell biological functions, while its activation is continuous in CAFs and cancer cells (Avalle et al., 2022; Johnson et al., 2018; Li H. et al., 2022; Yu et al., 2014). Inhibition of STAT3 has the advantage of targeting tumors while circumventing the killing of normal cells. Therefore, the development of new STAT3-targeted drugs may realize the dual targeting effect on CAF and lung cancer cells, and play the role of EGFR-TKI resistance reversal. Currently, the researches of STAT3 inhibitor are beginning to focus on the DNA binding domain, which is also the reason for the strong side effects (Huang et al., 2018; Son et al., 2017; Wu B. et al., 2021). Our previous study reported that inhibition of STAT3 in resistant lung cancer cells significantly increased the sensitivity of cells to gefitinib (Liu et al., 2021). Thus inhibiting STAT3 activation may reverse osimertinib resistance in NSCLC. In this study we found that LL1, a novel small molecule inhibitor of STAT3, blocked CAF-induced osimertinib resistance. Moreover, the results of xenograft demonstrated that CAF could significantly reduce the sensitivity of lung cancer cells to osimertinib, and LL1 combined with osimertinib could significantly inhibit CAF-induced osimertinib resistance *in vivo*. In terms of mechanism, CAF secretes MMPs through activation of STAT3, thereby promoting osimertinib resistance in non-small cell lung cancer (Figure 7). However, the molecular mechanism of CAF-induced osimertinib resistance needs further investigation.

## 5 Conclusion

This study offers valuable insights into osimertinib resistance in NSCLC, particularly in relation to CAFs in tumor microenvironment that contribute to osimertinib resistance. These findings suggest that targeting the CAF-mediated osimertinib resistance may hold promise as a potential therapeutic strategy for the treatment of resistance in NSCLC.

## Data Availability

The datasets presented in this study can be found in online repositories. The names of the repository/repositories and accession number(s) can be found below: http://www.proteomexchange.org/, PXD064318.
