# Supplementary material for: STAT3 mediates CAF-induced osimertinib resistance via regulating protein secretion in non-small cell lung cancer
Source: Front Pharmacol. 2025 Jul 9;16:1546491. doi: 10.3389/fphar.2025.1546491 (PMC12283998; doi:10.3389/fphar.2025.1546491)
Supplement: Supplementary file 1 [file Supplementaryfile1.docx]

**STAT3 mediates CAF-induced osimertinib resistance via regulating protein secretion in non-small cell lung cancer**

Xuchen Fan ^a, c, 1^, Sheng Wu ^a, c, 1^, Honglong Wu ^a, c^, Yingying Huang ^a, c^, Xuhui Tong ^c^, Meiling Yu^a, c*^, Zhe Liu^a, b, c*^

^a^ *Department of Pharmacy, The First Affiliated Hospital of Bengbu Medical University, Changhuai Road, Bengbu, 233000, Anhui, China*

^b^ *Anhui Province Key Laboratory of Respiratory Tumor and Infectious Disease, The First Affiliated Hospital of Bengbu Medical University, Changhuai Road, Bengbu, 233000, Anhui, China*

^c^ *School of Pharmacy, Bengbu Medical University, Donghhai Avenue, Bengbu, 233030, Anhui, China*

^1^ These authors contributed equally to the work.

^*^ Corresponding author. The First Affliated Hospital of Bengbu Medical University, Anhui, China. E-mail addresses: yumeiling409@sohu.com (M.L. Yu), liuzhe@bbmc.edu.cn (Z. Liu).

**Contents**

Supplementary Method and Materials……...…………………………………………….….3

Figure S1……………………………………………………………………………………..5

Figure S2……………………………………………………………………………………..6

Figure S3……………………………………………………………………………………..7

Figure S4……………………………………………………………………………………..8

Figure S5……………………………………………………………………………………..9

**Supplementary Method and Materials**

**Proteomics analysis**

Conditioned medium of CAF and tumor (about 50 mL) was collected, and then homogenized in 800 μL phenol extraction buffer, after that 1 mL saturated phenol with Tris-HCl (pH 7.8) was added. After several times shake, the mixture was kept at 4°C for 30 min. The upper phenolic phase was separated from the aqueous phase by centrifugation at 7100 rpm at 4°C for 10 min, transferred to a fresh tube and mixed with five volumes of pre-cold 0.1 M ammonium acetate-methanol. After being kept at -40°C overnight, the mixture was centrifuged at 12,000 rpm for 10 min at 4°C to pellet precipitated protein. For the wash step, the pellet was resuspended twice with pre-cold methanol and twice with ice-cold acetone. Following another round of centrifugation, dry at room temperature (about 5 minutes) and dissolve in the sample lysate at room temperature for 3-5 minutes. The solution was centrifuged at 4°C at 12000 rpm for 10min, the obtained supernatant was total protein solution. The total protein concentrations were quantified by bicinchoninic acid assay.

According to the measured protein concentration, take the same quantity protein from each sample, and dilute different groups of samples to the same concentration and volume. Add 25 mM DTT of the corresponding volume into the above protein solution to make the DTT final concentration about 5 mM, and incubate at 55°C for 30 min. Then add the corresponding volume of iodoacetamide so that the final concentration was about 10 mM, and place it in the dark for 15-30 min at room temperature. Then 6 times of the volume of precooled acetone in the above system to precipitate the protein, and place it at -20 °C for more than four hours or overnight. After precipitation, take out the sample and centrifuge at 8000 rpm for 10 min at 4 °C for collecting the precipitate. According to the amount of protein, add the corresponding volume of enzymolysis diluent to redissolve the protein precipitate, then the solutions were incubated for digestion at 37°C for 12 h. Finally, samples were lyophilized or evaporated after enzymolysis.

For TMT labelling, the lyophilized samples were resuspended in 30 μL 100 mM TEAB and Labeling reaction in a 1.5 mL Ep tube. 20 μL acetonitrile were added to TMT reagent vial at room temperature. The centrifuged reagents were dissolved for 5 min and mixed for centrifugation and repeat this step once. Then 10 μL of the TMT label reagent was added to each sample for mixing. The tubes were incubated at room temperature for 1 h. Finally, 5 µL of 5% hydroxylamine were added to each sample and incubated for 15 min to terminate reaction. The labeling peptides solutions were lyophilized and stored at -80°C.

RP separation was performed on an 1100 HPLC System (Agilent) using an Agilent Zorbax Extend RP column (5 μm, 150 mm × 2.1 mm). Mobile phases A (2% acetonitrile in HPLC water) and B (98% acetonitrile in HPLC water) were used for RP gradient. The solvent gradient was set as follows: 0~8 min, 98% A; 8~8.01 min, 98%~95% A; 8.01~30 min, 95%~80% A; 30~43 min, 80~65% A; 43~53 min, 65~55% A; 53~53.01 min, 55~10% A; 53.01~63 min, 10% A; 63~63.01 min, 10~98% A; 63.01~68 min, 98% A. Tryptic peptides were separated at an fluent flow rate of 300 μL/min and monitored at 210nm. Samples were collected for 8-54 minutes, and eluent was collected in centrifugal tube 1-15 every minute in turn. Samples were recycled in this order until the end of gradient. The separated peptides were lyophilized for mass spectrometry.

All analyses were performed by a Q Exactive HF mass spectrometer (Thermo, USA) equipped with a Nanospray Flex source (Thermo, USA). Samples were loaded and separated by a C18 column (15 cm × 75 µm) on an EASY-nLCTM 1200 system (Thermo, USA). The flow rate was 300 nL/min and linear gradient was 45 min (0~4 min, 8-11% B; 4~36 min, 11-45% B; 36~39 min, 45~100% B; 39~45 min, 100% B. mobile phase A = 0.1% FA in water and B = 0.1% FA in ACN). Full MS scans were acquired in the mass range of 350 – 1500 m/z with a mass resolution of 45000 and the AGC target value was set at 3e6. The 20 most intense peaks in MS were fragmented with higher-energy collisional dissociation (HCD) with collision energy of 32. MS/MS spectra were obtained with a resolution of 3000with an AGC target of 2e5 and a max injection time of 40 ms. The Q Exactive HF dynamic exclusion was set for 30.0 s and run under positive mode.

**Protein array**

Protein chips (GSH-CAA-440) were purchased from RayBiotech (USA). CAF and CAFKO cells were collected separately. The cells were lysed and the total protein concentrations were quantified by bicinchoninic acid assay. The slide chips were equilibrated at room temperature for 20-30 min and then placed in a vacuum desiccator or dried at room temperature for 1-2 h. Add 100µL of sample dilution to each well and incubated on a shaker at room temperature for 1 h to close the quantitative antibody chip. Draw off the buffer in each well. Add 90µl of sample to the wells, and incubate at 4°C overnight. The slides were washed using a Thermo Scientific Wellwash Versa chip washer. Centrifuge the detection antibody mixture tubes. Add 1.4 ml of sample diluent, mix well and centrifuge again quickly. Then add 80µl of detection antibody to each well and incubate for 2 h on shaker. Wash and centrifuge the Cy3-Streptavidin tubes, then add 1.4 ml of sample dilution, mix well and centrifuge quickly again. Add 80µl of Cy3-Streptavidin to each well and incubate with aluminum foil wrapped slides protected from light for 1 h on shaker at room temperature. After washing, fluorescence was detected using an InnoScan 300 Microarray Scanner.

**Bioinformatics analysis**

Proteins were analyzed for signifcant diferences in expression. Then, the clustering of diferential proteins, Gene Ontology (GO, https://www.geneontology.org/) enrichment, Kyoto Encyclopedia of Genes and Genomes (KEGG, https://www.kegg.jp/kegg/pathway.html) pathway enrichment were analyzed.

**Supplementary
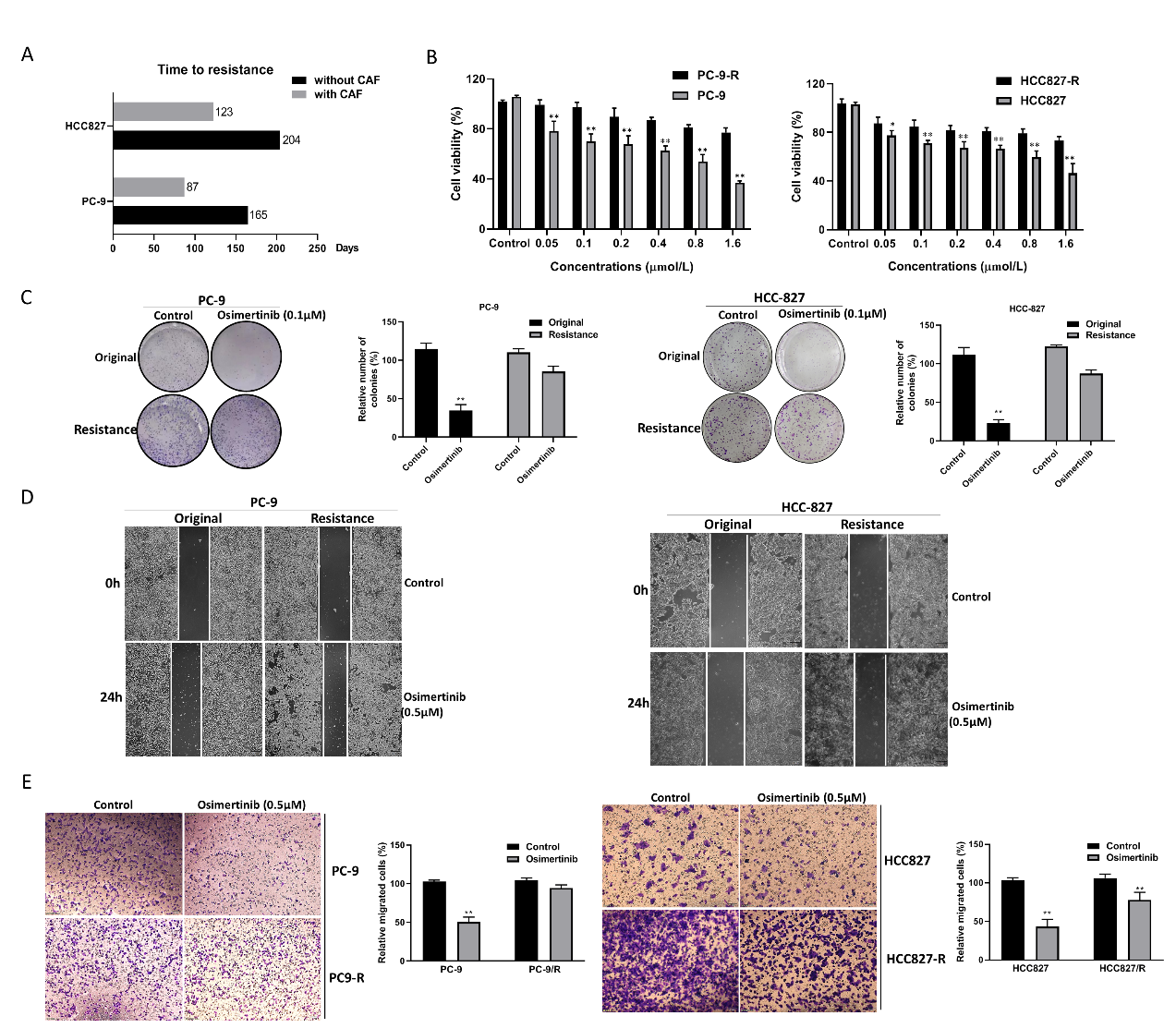
Fig. 1.** Osimertinib resistant PC-9 and HCC827 cells showed enhanced proliferation, invasion and migration.

**(A)** Effect of CAF on the time to induce osimertinib resistance in PC9 and HCC827 cells. **(B)** CCK-8 assay showed the proliferation of PC-9, PC-9 resistance, HCC827 and HCC827 resistance cells following the treatment of osimertinib. **(C)** Colony formation assay showed the single cell proliferation potential of PC-9, PC-9 resistance, HCC827 and HCC827 resistance cells following the treatment of osimertinib. **(D)** Wound healing assay showed the migration of PC-9, PC-9 resistance, HCC827 and HCC827 resistance cells following the treatment of osimertinib. **(E)** Transwell showed the invasion of PC-9, PC-9 resistance, HCC827 and HCC827 resistance cells following the treatment of osimertinib. Data are presented as mean ± standard deviation. *P* values were calculated using unpaired two-tailed Student’s t-test. ***P* < 0.01.

**
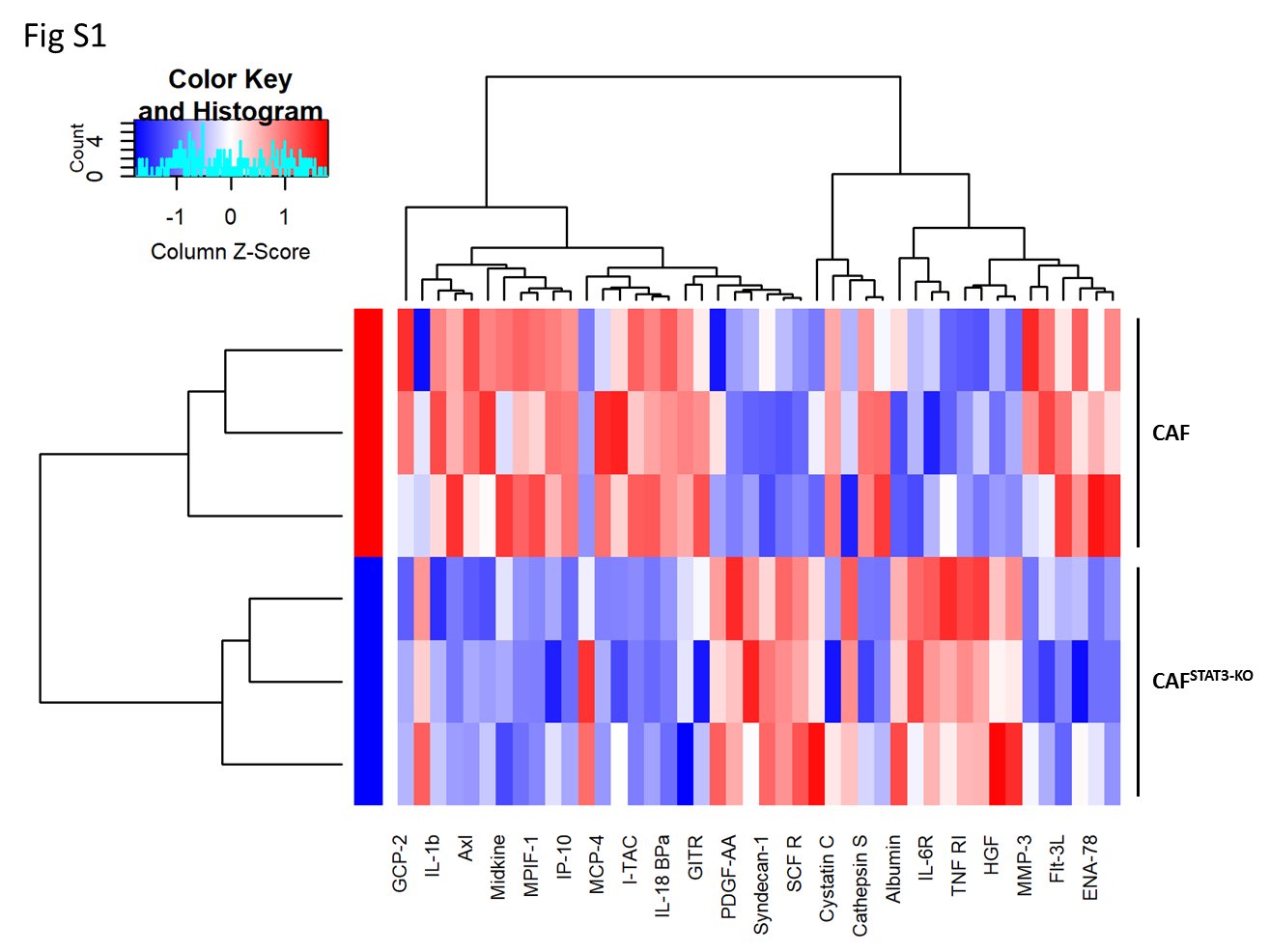
**

**Supplementary Fig. 2** Cluster heatmap of CAF and CAF^STAT3-KO^ by protein array.


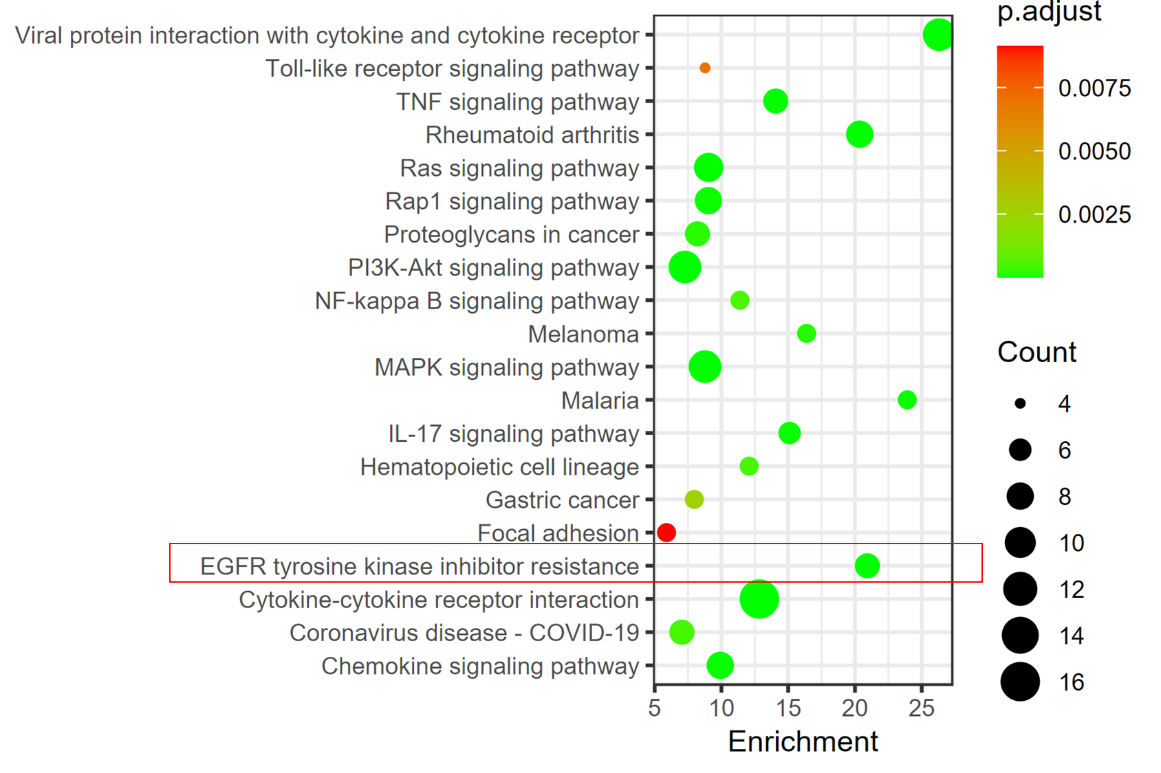


**Supplementary Fig. 3** KEGG enrichment analysis of CAF and CAF^STAT3-KO^ by protein array.


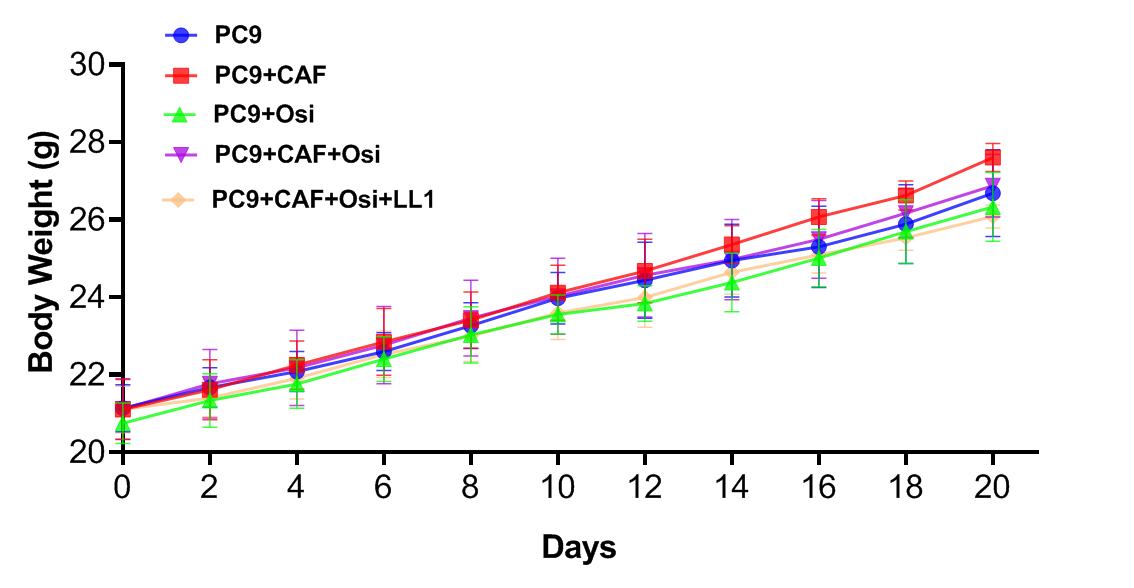


**Supplementary Fig. 4** The body weight of nude mice.


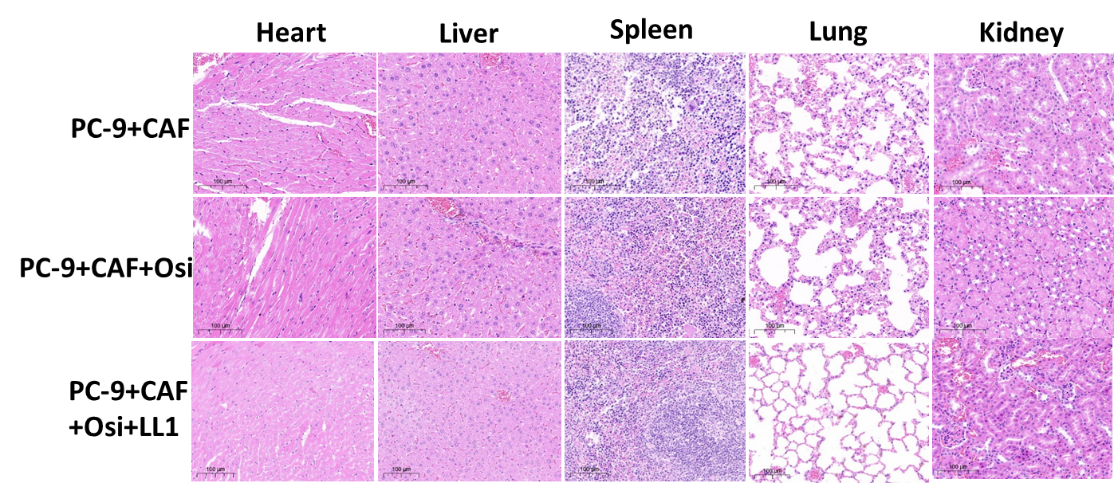


**Supplementary Fig. 5** H&E staining for heart, liver, spleen, lung, kidney.
